# Deconstruction and reconstruction of degrading effects in ultrasound imaging: aberration, multiple reverberation, and trailing reverberation

**DOI:** 10.1088/2057-1976/ae4d4d

**Published:** 2026-05-11

**Authors:** Danai E Soulioti, Rebecca M Jones-Sinnott, Masashi Sode, Francisco Santibanez, Gianmarco F Pinton

**Affiliations:** 1 Lampe Joint Department of Biomedical Engineering, North Carolina State University, Raleigh, NC 27695, United States of America; 2 The University of North Carolina at Chapel Hill, Chapel Hill, NC 27599, United States of America

**Keywords:** aberration, fullwave simulation, tissue brightness, reverberation, trailing clutter, acoustic propagation, abdominal ultrasound

## Abstract

Ultrasound image degradation in the human body arises from the propagation and reflection of acoustic waves in a complex acoustic environment. The heterogeneous distribution of soft tissue and the variation in acoustic properties distort the ultrasonic beam causing deterioration in image quality, including loss of resolution and contrast. Here, we establish a framework to construct images based on a separable (additive or multiplicative) representation of aberration, multiple reverberation, and trailing clutter. A separable approach enables high modularity and flexibility when generating quantitatively degraded image datasets. This framework provides the capability to generate images with quantitative levels of image degradation related directly to imaging physics, thus allowing for a flexible approach to augmentation techniques in ultrasound imaging datasets, as demonstrated in the included repository code. Experimentally calibrated abdominal simulations were performed in Fullwave2 by matching relevant imaging metrics, such as phase aberration, reverberation strength, speckle brightness, and coherence length, to experimental measurements. Then, simulations were performed to separate and characterize the different components of image degradation. Finally, these components were scaled and combined to construct quantitatively degraded image datasets. Reverberation is shown to be depth and brightness dependent, while aberration and trailing clutter are not. This general framework was tested for values in acoustic ranges that significantly, synthetically, and independently enhance or reduce these effects compared to the levels naturally occurring in the body. Identifying, quantifying, and modeling these differing and complex mechanisms of degradation can be used to develop and test rational approaches to overcome these degradation mechanisms to improve image quality, particularly for traditionally harder to image patients. Additionally, the framework to synthetically modify the effects of aberration, multiple reverberation, and trailing clutter is provided, allowing for the generation of augmented datasets with a wide range of degradation effects, based on imaging physics, to improve machine learning models.

## 1. Introduction

The heterogeneous acoustic properties of different tissue types, namely sound speed, density, and attenuation, distort and reflect the ultrasound wavefront as it travels to the target and as the echoes travel back to the transducer. In human abdominal imaging, especially, multiple layers of connective tissue and fat generate reverberation clutter and aberrate the beam to the point where diagnostic ultrasound imaging becomes difficult or impossible for some patients, requiring additional magnetic resonance imaging for the diagnosis of certain conditions Bohte *et al.* (2012). These factors usually occur in overweight and obese individuals, typically having a body mass index (BMI) higher than 30, who have thicker layers of subcutaneous fat and more fascial or connective tissue layers that support this additional fat Brahee *et al.* (2013). In this work, three medium-dependent degradation mechanisms–aberration, multiple reverberation and trailing clutter–and their relationship to imaging environment variables, such as depth and target brightness, are investigated.

Aberration occurs due to the inhomogeneity of the speed of sound in tissue which deviates from the constant value used in ultrasound beamforming (typically 1540m/s), distorting the ultrasound wave. The wave front distortion decreases image resolution and introduces noise that obscures the target of interest. There has been broad research interest aimed at mitigating or correcting aberration and a wide variety of techniques have been proposed to compensate for aberration and speed of sound deviations. Near-field correction techniques, for example, assume that the distortion can be approximated by a thin phase screen at or near the transducer surface Flax and O’Donnell (1988, 1989), Ng *et al.* (1994). However, even though this class of phase aberration correction techniques is relatively simple to implement, in many cases it results in limited improvements in image quality Trahey *et al.* (1990), Rigby *et al.* (2000), Dahl *et al.* (2006). Distributed aberration models and correction techniques have been developed based on the more physically realistic assumption that aberration occurs deep within soft tissue Fink (1992), Liu and Waag (1994), Thomas and Fink (1996). Assuming a constant aberrator thickness, real-time correction methods to correct for the wavefront distortion due to refraction have been explored in 3D Lindsey and Smith (2014). More recently, clinical implementations have used arrival-time correction to improve patient echocardiograms in 2D Måsøy *et al.* (2023) and 3D Måsøy *et al.* (2024). Using a matrix imaging approach, the response between transmitted and received focal spots can be decoupled Lambert *et al.* (2020, 2021). It has been shown that this approach can be used to establish a focusing quality criterion, correct for aberration, and quantify the single and multiple scattering components of the impulse response matrix  Lambert *et al.* (2020, 2021), Bendjador *et al.* (2021). Coherence-based methods have been proposed to estimate the average speed of sound as a function of depth as well as tomographic reconstruction techniques to estimate the spatially varying speed of sound distribution Ali *et al.* (2021, 2022, 2023), De La Torre *et al.* (2025).

Reverberations occur when an acoustic wave is reflected multiple times and it typically appears as clutter, or spurious ultrasound signal, that is overlaid on the underlying tissue image, thus resulting in significant degradation of image quality. Its effects on image quality are broad and can be characterized by loss in resolution and contrast. Reverberation clutter arises from multiple reverberations between layers of tissue with different properties, scattering from off-axis targets or side and grating lobes. Reverberation has been modeled previously using a pseudo-nonlinear approach, where experimentally-validated reverberation clutter is generated using linear simulation tools. This approach reproduces the characteristics of reverberation clutter with similar speckle statistics to experimental measurements and theory, but does not directly model the underlying multiple scattering physics Byram and Shu (2016). Along with aberration, reverberation is one of the main degrading mechanisms responsible for poor image quality; however, reverberation correction has been less investigated, perhaps due to the inherent difficulty of correcting multiple scattering events Dahl *et al.* (2012). Tissue harmonic imaging is perhaps the most widely used method that has been shown to improve image quality in the presence of clutter and it is used extensively in ultrasound imaging Spencer *et al.* (1998), Dahl *et al.* (2012). Imaging methods based on the short-lag spatial coherence (SLSC) of backscattered echoes have been proposed to reduce background clutter and increase signal-to-noise ratio  Lediju *et al.* (2011) and as an image quality metric Long *et al.* (2018a). Applications of this method, as well as its harmonic counterpart, have been shown during *in vivo* fetal imaging, where the contrast-to-noise ratio doubled for the latter Kakkad *et al.* (2015). Other post-processing approaches to reduce reverberation and off-axis clutter include algorithms such as aperture domain model image reconstruction (ADMIRE) beamforming, validated during *in vivo* fundamental and harmonic imaging scenarios Dei et al. (2018), Byram and Shu (2016), and machine learning approaches using neural networks Brickson *et al.* (2021), Jahren *et al.* (2023).

Inter-layer reflections that are transmitted in the direction of pulse propagation, instead of returning to the transducer surface, add a long, low-amplitude tail to the pulse, causing it to appear lengthened, resulting in additional clutter further away from the transducer and compromising resolution Dahl *et al.* (2012). This phenomenon is known as trailing clutter, and is often included in total reverberation clutter. While some techniques that reduce reverberation clutter also reduce trailing clutter, like harmonic imaging and SLSC, trailing clutter specific characterization and correction techniques are generally lacking from the literature and could require more investigation. In previous work, the role of phase aberration and reverberation as two major mechanisms in the degradation of ultrasound image quality were analyzed Pinton *et al.* (2011). It was also hypothesized that reverberation clutter from subcutaneous tissue layers limited the effectiveness of phase aberration correction techniques by corrupting the RF channel signals. Pinton *et al.* (2010, 2011, 2014). Recently, the effects of aberration, multiple reverberation, and trailing clutter have been isolated and characterized in the context of the medium-dependent resolution limits in super-resolution ultrasound imaging McCall *et al.* (2021) and transcranial imaging Soulioti *et al.* (2025).

Identifying, quantifying, and modeling these complex mechanisms of degradation are a critical component to develop rational strategies that can improve image quality. Specifically, understanding their separate effects on image quality and how they affect the image, like whether they affect deeper or brighter structures, is vital to understanding the imaging scenarios where these correction techniques are most necessary, like for deeper structures or higher BMI patients. The generation of an ultrasound image of the soft tissue in the body relies on the physics of acoustic wave propagation in heterogeneous media, which includes the effects of diffraction, reflection, scattering, frequency dependent attenuation, and nonlinearity. Simulations are an invaluable tool in characterizing and quantifying the relationship between wave propagation, human anatomy, and the resulting ultrasound image. Human abdomen models have been used in simulations in past literature Mast *et al.* (1997), Hinkelman *et al.* (1998), Mast *et al.* (1998) and were assessed using measurements of arrival time, wavefront distortion, and energy loss for different tissue types. The mechanisms of multiple reverberations and phase aberration in the abdomen have been described in previous work using Fullwave, a simulation tool that solves the non-linear fullwave equation using a finite differences in the time domain (FDTD) approach Pinton *et al.* (2009, 2010, 2011, 2014). Here, Fullwave2 Pinton (2021), an updated higher-order version of Fullwave that uses a FDTD approach on a scattered grid is used, which has been benchmarked with other ultrasound simulation tools Aubry *et al.* (2022). In this work, we introduce an experimentally calibrated platform that allows for the isolation of each component of degradation and its subsequent removal from or addition into a B-mode image. Multiple scattering is separated into two components, multiple reverberation and trailing clutter. A critical distinction between these two types of acoustic noise, or image degrading signal, is established based on the definition that multiple reverberation is not reflected by the target, whereas trailing clutter is reflected by the target. This general framework was calibrated to soft tissue measurements for specific transducers and imaging sequences for the human abdomen to characterize the depth and target brightness dependence for these different reverberating and aberrating environments. We show that this calibrated simulation method can span the parameter space of image degradation in an independent fashion, i.e., an individual component of degradation can be combined linearly based on wave propagation. It is shown *in silico* that the generation of realistic and propagation physics-based image degradation, that includes and exceeds ranges that would be encountered clinically in transabdominal imaging, is feasible. This includes cases such as distributed aberration with no reverberation or reverberation with no aberration, which would not be realizable experimentally. Such flexibility offers advantages in the generation of training databases for machine learning applications as well as the development of beamforming strategies for specific imaging scenarios. The code to augment aberration, reverberation, and trailing clutter by known amounts in post-processing were developed for use with any existing dataset and are included with this framework. This approach enables the creation of large and diverse machine learning datasets that mimic naturally occurring variations in image degradation, facilitating improved model training and more rigorous assessment of algorithm performance under systematically controlled degradation effects.

## 2. Methods

### 2.1. Separability of the sources of degradation

**Figure 1.**
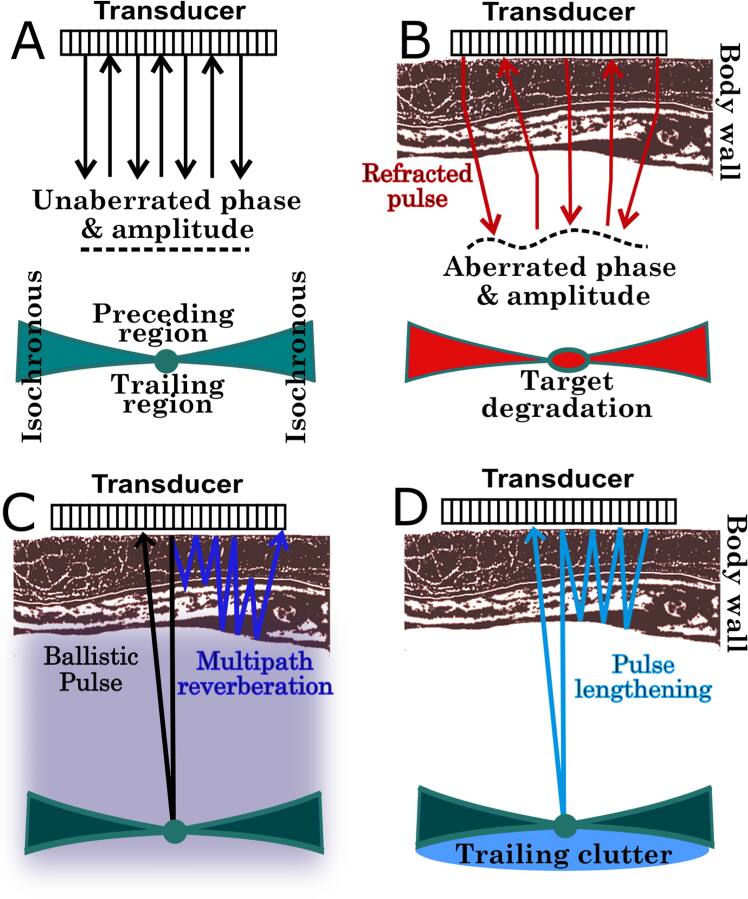
A, in a homogeneous medium, a typical PSF is characterized by three different regions, the pre-isochronous, trailing, and isochronous volume. In B, aberration degrades ultrasound focusing capability and compromises resolution. In C, multipath reverberation overlays acoustic noise to the echoes produced from the ballistic pulse, thereby reducing detectability. In D, the lengthening of the pulse results in the appearance of trailing clutter. Both reverberation and aberration depend on body wall anatomy.

The components of medium-dependent image degradation that affect an imaging pulse can be separated into three categories: (a) aberration of the phase and amplitude, (b) multiple scattering occurring near interfaces but appearing to be of distal spatial origin (reverberation clutter), and (c) multiple scattering that is co-directional with or spatially proximal to the imaging pulse (trailing clutter). This framework, summarized in figure 1, will be used to deconstruct and reconstruct ultrasound images.

A typical point-spread-function (PSF) of a point target in a homogeneous medium is shown in figure 1(A) for reference. The effects of aberration on a point target are illustrated in figure 1(B). Here, the term aberration is used to mean only a distortion of the phase and amplitude of the transmitted and reflected ballistic pulse, which is the pulse that travels directly to and from the target. The distortion of the pulse from heterogeneities in speed, index of refraction, and attenuation are all linear processes that are independent of pressure amplitude. The degradation arising from a given realization of aberration is thus independent of target brightness. In other words, if subjected to aberration alone, increasing the target brightness will not result in an improvement of resolution or contrast since the shape of the point spread function will remain the same.

In this model, the effects of reverberation clutter on image degradation are dependent on target brightness. This is illustrated schematically in figure 1(C), where an ultrasound wave can take two paths that arrive at the transducer surface at the same time. In the multiple reverberation path, the signal is reflected several times in the tissue layers (figure 1(C), blue line) but does not interact with the target. The amplitude of the multiple reverberation signal thus depends only on the reverberating medium. Multipath reverberation can occur during both forward and backward propagation. In the ballistic path (figure 1(C), black line), the wave travels directly to the target and is reflected back to the transducer surface. An increase in target brightness will result in a proportional increase in the reflected signal. Therefore, in this model of image degradation, increasing the target brightness improves the image quality since the ratio of the reflected ballistic pulse (signal) to the contemporaneous multipath reverberation signal (acoustic noise) increases.

Similar to aberration and unlike reverberation, in this model, the effects of trailing clutter on image degradation are independent of target brightness. This is illustrated schematically in figure 1(D), where the ballistic ultrasound pulse is lengthened by proximal scattering that travels in the same direction as the pulse. These scattering events have the effect of lengthening the original pulse therefore degrading its axial characteristics. Furthermore, since they travel with the pulse, they interact with and are reflected by the target. An increase in target brightness will result in a proportional increase in the reflected signal of both the ballistic pulse and its trailing clutter. Since the ratio of ballistic pulse (signal) to the trailing clutter (acoustic noise) is constant, its effect on image quality is independent of target brightness.

These medium-dependent sources of image degradation can be mapped to different locations in the PSF characterization of the imaging system Pinton *et al.* (2009). The PSF can be divided into three regions. The first is the isochronous volume, which for ultrasound imaging systems is typically bow-tie shaped. Signals within this volume have had time to travel from any point on the transducer surface to the target and back again to any point on the transducer surface. In the absence of reverberation or multipath propagation, all the PSF energy must be contained within this volume. Phase and amplitude aberrations will distort the shape and amplitude distribution within the isochronous volume. Signal in this region is also affected by multiple reverberation paths. The second region, referred to as the pre-isochronous volume, is spatially situated above the isochronous volume and corresponds to values in time that precede arrival with respect to the isochronous volume. Energy in this location of the PSF can physically arise from multiple reverberation paths that are shorter than the ballistic isochronous paths. The third region, the post-isochronous volume, trails the isochronous volume and corresponds to times that follow the arrival of the target signal. Signals that occur in the trailing region can arise from multiple reverberation paths that are longer than the ballistic isochronous path, or from trailing clutter.

**Figure 2.**
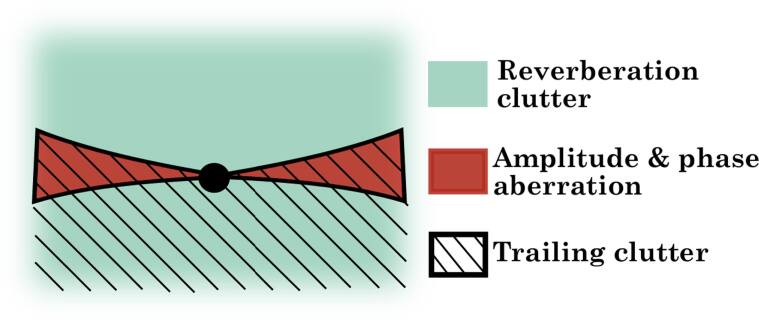
Illustration of all three medium dependent degradation effects on a point spread function.

The relationship between the areas of the PSF and the medium-dependent sources of degradation are illustrated in figure 2. The isochronous volume can be affected by all three sources of degradation, i.e., aberration, trailing clutter, and reverberation clutter. The pre-isochronous volume only admits energy from multiple reverberation paths that have not reached the target. The post-isochronous volume can contain energy from multiple reverberation paths and from trailing reverberation paths. Trailing clutter, which by definition represents a lengthening of the original transmitted pulse, therefore cannot occur in the pre-isochronous volume. The location of these different sources of degradation within the PSF are a key component that allows for the characterization and separability of the medium-dependent effects. It also allows for independent control of the three sources of image degradation when reconstructing synthetic ultrasound images.

Point spread functions are not the only way to describe reverberation and aberration, and *ex vivo* experiments offer additional flexibility for experimental and simulation characterization. Here, a porcine abdominal phantom in a water tank was used to measure characteristics of the wave propagation physics, aberration values, reverberation curves, speckle brightness, and coherence curves, and then appropriately calibrate the acoustic maps in the simulation tool. A C5-2v (ATL Ultrasound Inc, Bothell, WA) abdominal imaging transducer and a Vantage 256 ultrasound research scanner (Verasonics, Inc., Kirkland, WA) were used to capture RF channel signals for all experiments in this work. Transmits were performed with a 3.7 MHz 2-cycle focused wave emission in both experiment and simulations. Under the abdomen, the degassed water provides an anechoic region. Since no reflections occur in water, any signal measured by the imaging system that is spatially attributed to the water region must temporally correspond to multiple reverberations within the abdomen. This separability allows the characterization of reverberation magnitude and its depth-dependence. Then, liver tissue was placed under the abdominal wall to characterize its brightness and coherence values. To characterize the distortion from aberration, the liver was removed and replaced with a bright wire target reflector. The cylindrical wave generated by this target was characterized by measuring the distortion of the pulse received at the transducer surface. These experimental measurements of aberration, reverberation, speckle brightness, and coherence curves and their use in calibrating simulation tools are described in detail in the following sections 2.4, 2.5, 2.6 and 2.7. Section 2.2 focuses on the generation of the input maps to the simulation tool. A github repo to artificially augment aberration, reverberation, and trailing clutter in post-processing on any existing dataset, allowing for the generation of larger datasets with varying degradation values for machine learning applications, is included and described in the Appendix.

### 2.2. Simulations

**Figure 3.**
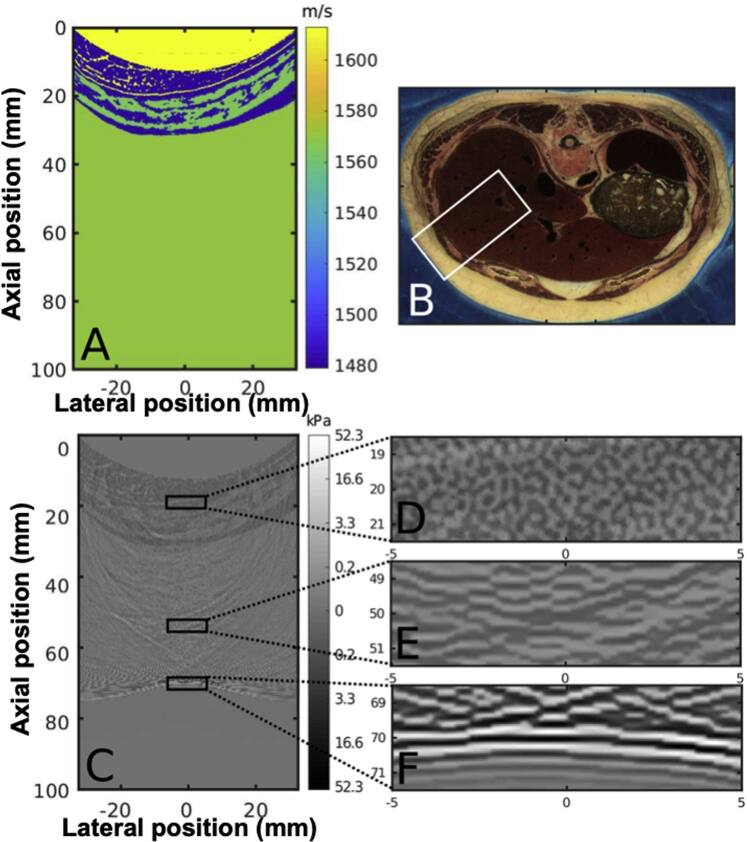
A, Example of an input sound speed map for Fullwave2 simulations, as segmented and processed from the selected portion of B, the visible female abdominal dataset. In C, the result of a wave propagating through the map (A) is shown. Highlighted are different characteristic regions of propagation: D is the highly incoherent portion of the wave as it travels through the abdominal wall. As the wave enters the pre-isochronous region in E, coherence increases as it approaches its natural focal zone. In F, the wave has reached its maximum coherence value at the focus.

Simulations were performed using Fullwave2 Pinton *et al.* (2009), Pinton (2021) with the corresponding open source code available at the following repository: https://github.com/gfpinton/fullwave2.git. Simulations were performed using a heterogeneous tissue model of a human abdominal layer derived from photographic cadaveric cryosections  Ackerman (1998), Pinton (2020, 2021). The simulation region (figure 3(A)) is derived from the subset of the visible human data depicted by the white inset box in figure 3(B). Different tissue types were segmented Pinton (2020) and assigned one of three tissue types: fat, muscle, or connective tissue. Each type is given the corresponding previously tabulated values for speed of sound, density, non-linearity, and attenuation (figure 3(A)) Goss *et al.* (1978). The abdominal maps have a resolution limit of 330 *μ*m and therefore cannot resolve cellular and extracellular structures that generate speckle in ultrasound images. Note that sub-resolution scatterers are also not visible in the acoustic map images due to their size. These sub-resolution, pixel-sized scatterers were modeled and calibrated to simulate the speckle-generating scattering physics of tissue. The scatterer properties and brightness were calibrated using methods that are discussed in detail in subsection 2.6, which is dedicated to this topic. A Gaussian smoothing kernel with a standard deviation of 1.5 pixels was performed on the tissue maps to reduce discretization errors from boundary segmentation. The C5-2v transducer has a curved aperture as shown is figure 3(A) by the yellow arc portion. A linear deformation was used to model the pressing of the transducer into the abdomen. In the simulation, the transducer surface is located just above the interface between the yellow arc and the soft tissue beneath it. All simulations were performed on a Linux Fedora 25 (v.4.10.13-200.fc25.x86 64) system running Intel Xeon^®^ E5-2630 v4 Processors at 2.20 GHz. The simulation code was written in C and post-processing was performed in MATLAB. Every simulation for each individual emission case has an approximate duration of 30 min. The simulation grid size has a spatial resolution of 34.7*μ*m and the time steps have a duration of 5.4 ns. Simulations were beamformed using delay-and-sum beamforming, with all figures normalized to their maximum value, allowing for the relative effects of degrading mechanisms to be compared, with the exception of reflectivity comparisons.

The output of the simulations is the pressure as a function of space and time for the entire physical domain. Figure 3(C), shows a snapshot in time of the pressure of a focused wave that has propagated 70 mm through the abdomen and into the liver. For illustration purposes, the pressure is overlaid on the input sound speed map. Three distinct regions of behavior can be identified within this wave field. The inset in figure 3(D), which shows the wave field in the abdominal wall, is highly incoherent. In this time snapshot, the signal is trapped in the abdomen and has undergone a large number of scattering events, which removes any preferred directionality or coherence. In the homogeneous region beneath the abdominal wall, shown in figure 3(E), the wave-field has some coherence along the lateral axis since it has traveled preferentially along the axial direction. By contrast, the inset in figure 3(F) shows the ballistic pulse with its characteristic curvature which allows it to focus. The energy in this pulse carries the information necessary for imaging. A slight distortion of the profile is visible. There is also energy immediately trailing this ballistic pulse. The trailing signal is partially coherent with a principal coherence direction orientated approximately along the lateral or y-axis. The structure of this wave-field can be related to the regions of the PSF. The isochronous volume is composed primarily of the ballistic pulse. The reverberation clutter is composed of signal trapped in the abdomen. Finally, trailing clutter is composed of the signal following the ballistic pulse.

### 2.3. Isolating and reconstructing components of degradation

Acoustic maps were modified *in silico* to linearly isolate image degrading effects into their individual components. These maps were designed to remove aberration while preserving reverberation or, conversely, to remove reverberation while preserving aberration Pinton *et al.* (2009). Isovelocity maps were generated to have a homogeneous speed of sound and a modified density that maintains the original impedance of the tissue. The original reference impedance as a function of space, *Z*_ref_, is defined as

\begin{eqnarray*}{Z}_{{\mathrm{ref}}}(x,y)={\rho }_{{\mathrm{ref}}}(x,y){c}_{{\mathrm{ref}}}(x,y)\end{eqnarray*}

where *ρ*_ref_ is the original density map and *c*_ref_ is the original speed of sound map. Then, if the isovelocity map has a desired constant or homogeneous speed of sound value *c*_0_ for all of space, i.e.,

\begin{eqnarray*}{c}_{{\mathrm{iv}}}(x,y)\equiv {c}_{0}\end{eqnarray*}

then, to maintain the same impedance distribution as in the reference case, the density must be

\begin{eqnarray*}{\rho }_{{\mathrm{iv}}}(x,y)=\frac{{c}_{0}}{{Z}_{{\mathrm{ref}}}(x,y)}\end{eqnarray*}

To generate isovelocity images, the sound speed maps in the simulation were set to be constant at a reference value of *c*_0_ = 1570 m/s, which corresponds to the average speed of sound throughout the map. The attenuation and nonlinearity maps remained unchanged.

A similar process was used to generate isoimpedance maps. To maintain the same speed as in the reference map for a desired given homogeneous impedance value, *Z*_0_, that is constant for all of space,

\begin{eqnarray*}{Z}_{{\mathrm{ii}}}(x,y)\equiv {Z}_{0}\end{eqnarray*}

where the velocity remains unchanged, i.e., *c*_ii_ = *c*_ref_, and the isoimpedance density, *ρ*_ii_ must be:

\begin{eqnarray*}{\rho }_{{\mathrm{ii}}}(x,y)=\frac{{Z}_{0}}{{c}_{{\mathrm{ref}}}(x,y)}\end{eqnarray*}

To generate isoimpedance images, the impedance maps in the simulation were set to be constant at a reference value of *Z*_0_ = 1.67 MRayl, which corresponds to the average impedance throughout the map. The attenuation and nonlinearity maps remained unchanged.

This simulation framework was also used to reconstruct images with known and modulable amounts of image degradation. In the isovelocity case, for example, the speed can be maintained at a constant value *c*_0_, but the density can be artificially increased or decreased to change the amount of reverberation without adding aberration. To achieve this, the density map, *ρ*_iv_(*x*, *y*), is modulated by desired factor, *γ*, of the impedance mismatch

\begin{eqnarray*}{\rho }_{{\mathrm{m}}}(x,y)={\rho }_{0}+\gamma ({\rho }_{{\mathrm{iv}}}(x,y)-{\rho }_{0})\end{eqnarray*}

where *ρ*_0_ is the equilibrium density around which the impedance mismatch is determined, e.g., the mean density as function of space. Note that in this case, the speed map remains constant as a function of space.

Similarly, the isoimpedance map can be modulated to introduce changes in the speed map that modify the aberration without affecting the reverberation magnitude. To achieve this, the excess speed of sound is multiplied by the scalar *ζ*

\begin{eqnarray*}{c}_{{\mathrm{m}}}(x,y)={c}_{0}+\zeta ({c}_{{\mathrm{ii}}}(x,y)-{c}_{0})\end{eqnarray*}

where *c*_0_ is the equilibrium speed of sound around which the excess speed of sound is determined, e.g., the mean speed as function of space. In this case, the density is determined by the fact that the impedance is a constant as a function of space.

Equations (6) and (7) are formulated in a way that only allows independent modifications of the acoustic maps, i.e., either isoimpedance or isovelocity configurations. However, these linear operations can be combined to produce generalized maps that blend features of the isoimpedance maps and the isovelocity maps. This general modification allows for a continuous modulation of the quantity of reverberation or aberration. This can be achieved by calculating a new effective impedance based on the speed calculation in equation (7). The effective impedance based on this modified speed is expressed as a perturbation of a mean scalar impedance, i.e.,

\begin{eqnarray*}{Z}_{m}(x,y)={\rho }_{{\mathrm{ref}}}(x,y){c}_{m}(x,y)={Z}_{0}+{Z}^{{\prime} }(x,y)\end{eqnarray*}

Then, the modulation of the reverberation occurs based on a scaling by *γ* of the excess impedance, ${Z}^{{\prime} }$, and the updated speed map, *c*_*m*_,

\begin{eqnarray*}{\rho }_{m}(x,y)=\frac{{Z}_{0}+\gamma {Z}^{{\prime} }(x,y)}{{c}_{m}(x,y)}\end{eqnarray*}

Here, the parameter *γ* retains its physical meaning as described in equation (6). For example, when *γ* = 0, equation (9) is equivalent to equation (6) and the maps represent a pure isoimpedance modification. Similarly, in the case when *ζ* = 0, then *c*_*m*_ = *c*_0_, which represents a pure isovelocity case. In the case where *γ* = 0 and *ζ* = 0, the maps are completely homogenized to the values of *c*_0_ and *ρ*_0_. In the case where *γ* = 1 and *ζ* = 1, the maps return to their original values *c*_ref_ and *ρ*_ref_. Values of *γ* and *ζ* in between 0 and 1 will have a partial homogenization effect. Values of *γ* and *ζ* larger than 1 will exaggerate the heterogeneities. In summary, equations (7), (8) and  (9) describe the modifications of speed and density to artificially increase or decrease the effects of aberration and reverberation by known amounts within the same set of acoustic maps.

Changing the acoustic maps results in modification of the total reverberation, i.e., both the reverberation clutter and the trailing clutter incur a modification. Even though these two types of reverberation have a different impact on image quality, they arise from the same fundamental interaction with the acoustic medium and the structure of its impedance. To separate trailing clutter from reverberation clutter, an additional isoimpedance scenario, previously described here Pinton *et al.* (2009), was used. This case requires two simulations. In the first simulation, the entire acoustic field is modeled. For example, for a point spread function simulation, the point spread function is determined in the case where the point target at the focus is present. In the second simulation, the imaging target or targets are removed and only the clutter generating structures are retained. For example, for the point simulation, the target at the focus is removed but the abdomen is left in place. In this manner, the signal received by the transducer cannot be reflected from the focal location but can only be from multiple reverberation in the abdominal wall. Subtracting the latter from the former then results in removing the effects of only multiple reverberation, whilst preserving trailing clutter and aberration. This offers the ability to separate trailing clutter from reverberation clutter.

### 2.4. Aberration RMS values

Transmission measurements in a porcine phantom were performed to quantify the phase aberration. The phantom was comprised of a 20 mm thick porcine abdomen that was immersed inside a water tank kept at room temperature. This abdomen included a layer of depilated skin, followed by fat and muscle tissue. Connective tissue was also found throughout the abdominal layers. No liver was introduced in this experiment. The C5-2v transducer was placed directly above and in contact with the abdomen. Phase aberration is often characterized by measuring the amplitude of the arrival time fluctuations and the spatial variation of wavefront distortion along the array aperture Trahey *et al.* (1991), Liu and Waag (1995). The amplitude of these fluctuations is measured with the root-mean-square (RMS) value, which characterizes the strength of the phase aberration. This definition is also used in this work. The arrival time profiles for an aberrated pulse as well as a reference pulse in a homogeneous medium are calculated by cross-correlation between the radiofrequency waveforms from adjacent elements using the following equation:

\begin{eqnarray*}{\rho }_{n}=\frac{{\sum }_{j=1}^{k}({X}_{j}-{X}_{m})({Y}_{j}+n-{Y}_{m})}{{\left[{\sum }_{j=1}^{k}{({X}_{j}-{X}_{m})}^{2}{\sum }_{j=1}^{k}{({Y}_{j}+n-{Y}_{m})}^{2}\right]}^{\frac{1}{2}}}\end{eqnarray*}

where *ρ*_*n*_ is the calculated correlation coefficient between column k of data received at element X and the corresponding column of adjacent element Y displaced by n samples, with the mean value of X and Y being *X*_*m*_ and *Y*_*m*_, respectively. Once the arrival time profiles are calculated for both the aberrated case and the homogeneous case for the same array and emission, the root mean square difference of the two multiplied by the sampling period will yield a temporal measure of aberration, typically reported in ns.

To obtain the profiles for the RMS calculation described above, a thin wire was placed perpendicular with respect to the long axis of the transducer, thus acting as a point target, and its backscattered echo through the abdomen was recorded. This was compared against the same wire target at the same depth from the transducer in a water tank without any tissue (homogeneous medium), which served as a reference. Using the same transducer at the same frequency, Mast *et al.* Mast *et al.* (1997) performed water tank measurements using similarly sized porcine tissue. They reported aberration values in a range of 39-103 ns. In this work, where a highly aberrating model is used, the RMS value of the aberration for the experiment was calculated at 143.7 ns, whereas for the simulations, the RMS value was calculated at 146 ns.

### 2.5. Reverberation curves

**Figure 4.**
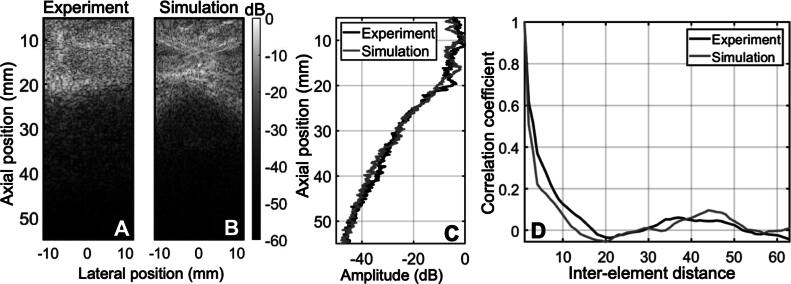
A, Experimental B-mode of pork belly immersed in water using the C5-2v probe. B, simulated B-mode of the visible human dataset used for calibration to experimental data. B-modes are normalized with respect to the maximum of each image. C, comparison of experimental and simulation reverberation curves for the first 60 mm of depth. D, coherence plots comparison for simulation and experiment at the focal depth (50 mm), in the center of the transducer laterally, plotted by element number, where the inter-element distance is 0.508 mm.

The same phantom described above without a wire target was used to measure the reverberation ring-down in the area beneath the abdominal wall. The liver was also not included in this configuration. Any signal measured in the water beneath the abdominal wall would necessarily be a result of multi-path reverberation since there are no reflective structures under the abdomen. Reverberation curves were acquired by laterally averaging the beamformed RF data and plotting them as a function of depth. The same process was followed for both experiments and simulations. In figures 4(A) and (B), the B-modes for both experiment and simulation are shown, respectively. The thickness of the abdomen is roughly the same at approximately 20 mm, with localized reverberation apparent until roughly the depth of 40 mm. The two B-modes were beamformed using a conventional delay-and-sum algorithm for a uniform beamforming speed of 1540 m/s. As expected, after 20 mm there is no tissue, yet there is still signal in the B-mode images due to reverberation, which persists for lengths of time that are coincident with reflections from depths greater than 20 mm. Figure 4(C) shows the reverberation curves for both experiment and simulation cases derived from images A and B, laterally averaged and displayed as a function of depth, which demonstrates an agreement between the two cases.

### 2.6. Speckle brightness

The liver, which is a relatively acousticly homogeneous organ, was used to calibrate the speckle brightness in simulations. To estimate the mean B-mode brightness value for the focal region, a piece of porcine liver was inserted under the abdomen. The liver and abdomen together spanned a total depth of 6 cm and it was long enough to cover the width of the transducer, which is approximately 3 cm. In simulations, the speckle statistics and tissue brightness levels were modeled using a random distribution of pixel-sized scatterers, which are characterized by a variable number of scatterers per resolution cell and a variable acoustic impedance. Simulations were performed for different scatterer characteristics until a satisfactory match between experimental and simulation values was achieved. The final calibrated speckle brightness values matched for the experiment (−43 dB) and the simulation dataset (−43.2 dB) for simulations with a density of 18 scatterers per resolution cell. Anechoic lesions were simulated with a field of randomly distributed sub-resolution scatterers inserted in the acoustic maps to model a uniform liver. In this field, three circular anechoic lesions were introduced at depths of 40, 60, and 80 mm by removing all scatterers within a radius of 5 mm. The simulated imaging sequence was extended to model 64 focused transmit-receive events. Each event directs the wave to a different focal location laterally in a sector scan format that follows the curvature of the C5-2v probe. The 64 individual foci were placed at intervals of beamwidth/2, corresponding to 173 *μ*m for the modeled transducer. One line per transmit was used for beamforming.

### 2.7. Coherence curves

The spatial coherence between two discrete signals received from an ultrasound transducer array can be estimated by a normalized correlation function, as previously described in equation (10) for a discrete time index of j and a temporal correlation window bound by k. Spatial coherence for the experimental and simulated data during the calibration process was measured by correlating the signals received by the transducer as a function of inter-element distance, or lag Pinton *et al.* (2014).

\begin{eqnarray*}\rho ({\mathrm{\Delta }}x)=\frac{1}{K-m}\displaystyle \sum _{i=1}^{K-m}\rho ({x}_{i},{x}_{i+m})\end{eqnarray*}

Here, K is the number of elements in the transducer and *m* is the inter-element distance, where the pitch is 0.508 mm for the C5-2v probe. This coherence curve is shown in figure 4(D). The correlation coefficients are calculated for a single full-aperture focus at 5 cm of depth and in the lateral midline, where liver tissue is present for both the experiment and simulation. Lag-one coherence (LOC), introduced by Long *et al.* Long *et al.* (2018b), can also be used as an image quality metric and was found to be correlated to traditional image quality indicators such as CNR. LOC leverages the spatial coherence between nearest-neighbor array elements to provide a local measure of thermal and acoustic noise. LOC can be calculated by setting *m* to be 1 in equation (11). LOC was measured to be 0.95 for the experiment and 0.96 for the simulation.

## 3. Results

### 3.1. Deconstruction of image degradation

#### 3.1.1. Contribution of aberration, reverberation, and trailing clutter to PSF degradation

**Figure 5.**
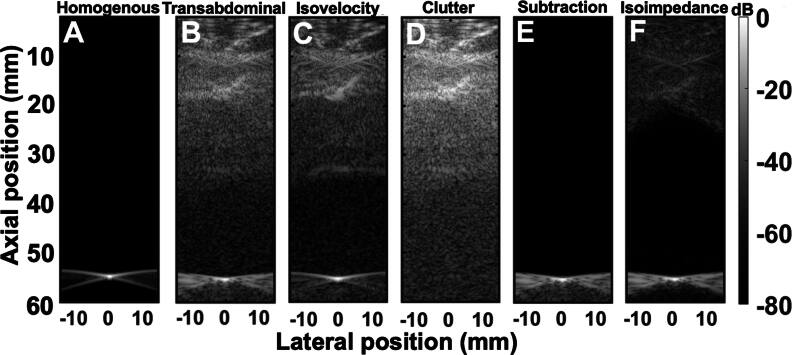
A-F, B-mode images for a target at 55 mm from the transducer normalized by the maximum amplitude of the homogeneous case. A single transmit was used with apodization on half the transducer elements. A, is the homogeneous B-mode, B is the heterogeneous B-mode through the abdomen, C is the isovelocity B-mode. D is the heterogeneous B-mode in the absence of the target. E is the isoimpedance B-mode through clutter subtraction, where D has been subtracted from B, whereas F is the isoimpedance B-mode through setting the isoimpedance to be constant.

PSF simulations were performed for six different cases as shown in figures 5(A)–(F). A scatterer was placed 55 mm away from the transducer in a homogeneous medium for reference, shown in figure 5(A), and under an abdominal section, producing the heterogeneous case shown in figure 5(B). The isovelocity B-mode, created using equation (6) where the sound speed is constant, is shown in figure 5(C). Clutter can be seen throughout the whole PSF. Figure 5(D) is the heterogeneous B-mode with the point target removed, or clutter image, and is subtracted from figure 5(B) to produce the isoimpedance via subtraction B-mode shown in figure 5(E). Figure 5(F) is the isoimpedance B-mode produced by setting impedance to be constant and equal to the homogeneous values, as decribed in equation (7). The isoimpedance and clutter subtraction simulations show a distorted isochronous volume, with trailing clutter preserved in the isochronous and post-isochronous volume of the clutter subtraction case. Details on how the isovelocity and isoimpedance B-modes are derived are given in section 2.3.

**Figure 6.**
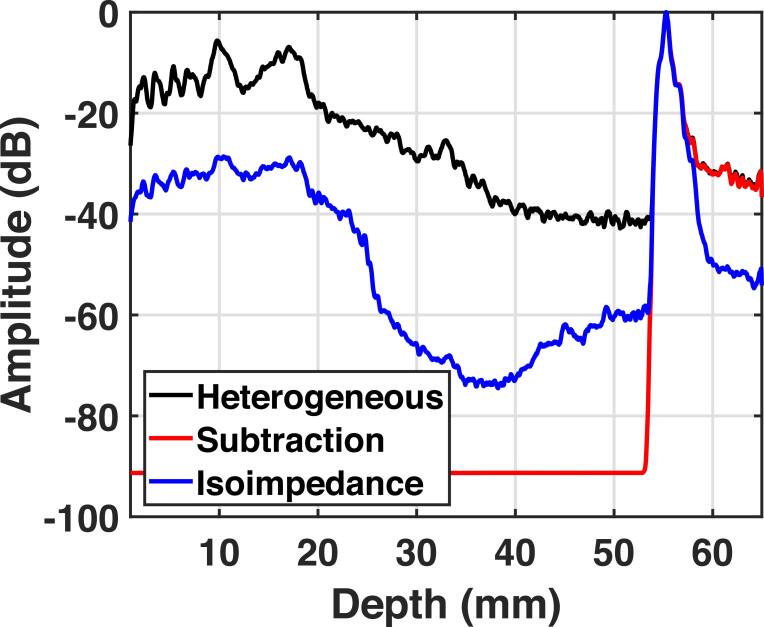
Reverberation curves for the heterogeneous, clutter subtraction, and isoimpedance B-modes, demonstrating the differences in the trailing and pre-isochronous regions for the two isoimpedance methods.

In the isovelocity case (figure 5(C)), the shape of the isochronous volume appears to be almost fully restored to its homogeneous state, even though clutter still degrades the target. In figure 5(E), the shape of the isochronous volume remains degraded even though reverberation clutter is completely removed. Note that, as expected, trailing clutter remains in the area under the PSF in figure 5(E). In the isoimpedance case, the reverberation and trailing clutter are significantly reduced, although not perfectly eliminated. This is a limitation of the numerical implementation. Plots of the signal magnitude, shown in figure 6, demonstrate that the reverberation subtraction method reduces reverberation clutter down to the numerical noise floor (93 dB), whereas the isoimpedance method reduces reverberation and clutter by 20-35 dB, depending on the depth, and reduces trailing clutter by 20-25 dB.

The three PSF regions were manually masked, with the isochronous volume mask including the ‘bow-tie’ shape. The mean amplitude of each of these three regions, pre-isochronous, trailing, and isochronous, is shown in table 1. For the pre-isochronous regions, inserting the abdomen resulted in a 31 dB increase compared to the reference homogeneous case. Removing phase aberration had little effect, decreasing the amplitude by an additional 3 dB. Removing reverberations via setting impedance to be globally constant resulted in a 15 dB increase from the original homogeneous value. Subtracting the background clutter has the greatest effect on the pre-isochronous region, increasing the amplitude by 8 dB compared to the original value in the homogeneous case. The amplitude of the isochronous region remains constant for all reverberation manipulations but is significantly restored when removing phase aberration, decreasing by 17 dB from the heterogeneous value.

**Table 1.** Average magnitudes (dB) of the three PSF regions relative to the main lobe for a scatterer at 55 mm.

	pre-isochronous	Trailing	Isochronous
Homogeneous	−91	−93	−60
Heterogeneous	−60	−50	−32
Clutter subtraction	−82	−50	−32
Isovelocity	−63	−52	−49
Isoimpedance	−76	−68	−32

The addition of the abdomen in the heterogeneous case increases the trailing region amplitude by 43 dB, compared to the original value of -93 dB for the homogeneous case. The trailing region amplitude is restored closest to its reference value for the isoimpedance case at -68 dB. Removing multiple reverberations via subtraction of clutter as well as removing phase aberration does not significantly affect the amplitude of the trailing region as compared to the heterogeneous case, with its values remaining at -50 and -52 dB, respectively. The effects of removing reverberation, described above, can be seen schematically in figure 6 for the isoimpedance and isoimpedance via clutter subtraction methods as compared to the heterogeneous case. As also shown in table 1, for the isoimpedance via subtraction case, the mean amplitude of the image up to the depth of the target drops to the lowest level comparable to the homogeneous case but there is no difference in the amplitude of the trailing region. For the isoimpedance case, the reduction of the amplitude of the trailing region is significant. However, the residual reverberation observed within the abdominal tissue layer indicates imperfect cancellation of reflections in the simulation tool, likely caused by small numerical errors at complementary speed/density boundaries arising from the staggered grid implementation. These errors are amplified by the second order derivative, which generates spurious reflections.

#### 3.1.2. Depth dependence of aberration and trailing clutter

**Figure 7.**
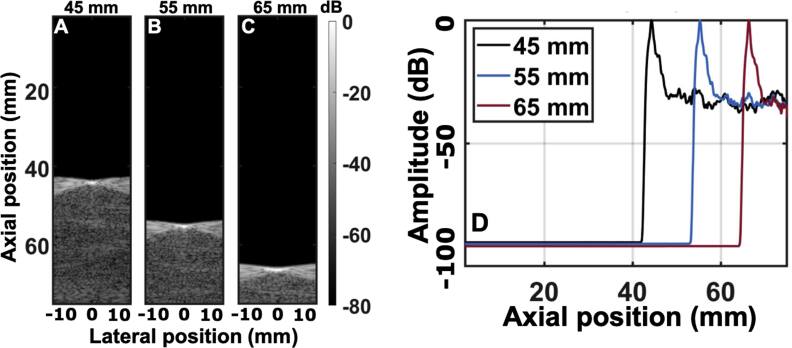
Isoimpedance via clutter subtraction B-modes for A, a point target at 45 mm in depth, B, a target at 55 mm, and C, a target at 65 mm. D, axial amplitude as a function of depth for B-modes A-C.

To determine the dependence of phase aberration on target depth, the same simulation setup through the human abdomen was used for targets placed at 45, 55, and 65 mm, with a single pulse focusing at the depth of each target. The effects of multiple reverberation were removed from the RF data via clutter subtraction before performing the RMS calculations to minimize confounding variability not stemming from phase aberration. To evaluate differences in depth, the relative change in RMS value was compared for the different depths. The B-mode images for these three cases described are shown in figures 7(A)–(C). For the three different depths, the RMS values of aberration were found to be 143, 130 and 139 ns RMS, respectively. These small variations show that there are not large differences in time delay errors due to aberration at these different imaging depths. While the magnitude of the time delay errors is similar, their spatial profiles are expected to differ due to spatially varying aberration patterns.

Trailing clutter is a degradation or lengthening of the transmit pulse that, based on the previously described physical model, is hypothesized to be independent of target depth. The axial amplitude as a function of depth (figure 7(D)) includes the effects of aberration, which are isolated in the isochronous volume, i.e., the peak for each PSF, and trailing clutter, which occurs after the peak. The clutter subtraction operation has removed the multipath signal. For all three depths, the trailing clutter signal remains relatively constant around -27 dB from the PSF peak. In other words, the trailing clutter magnitude is not a function of depth post-body wall.

#### 3.1.3. Effect of brightness on reverberation

**Figure 8.**
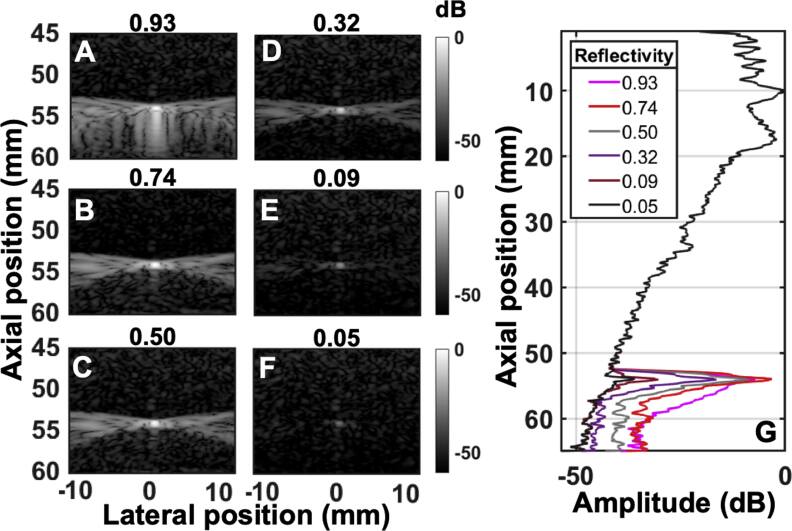
B-mode images for varying target reflectivities with respect to the background of: A, 0.93 B, 0.74 C, 0.50, D, 0.32 E, 0.09 F, 0.05, all plotted on the same color axis. G, reverberation curves for the different target reflectivity cases, demonstrating a trend of increasing trailing clutter amplitude with target reflectivity, with local variations between conditions.

We hypothesized, based on the propagation physics, that target characteristics in the focal region, such as brightness, do not affect the magnitude of multiple reverberation, which depends only on the properties of reverberating structures closer to the transducer, in this case the abdomen. This is unlike the ballistic pulse or trailing clutter whose magnitudes are proportional to target brightness. Varying the target brightness or the impedance mismatch between sub-resolution scatterers and the background tissue illustrates how the difference in target reflectivity affects image degradation mechanisms, especially in terms of reverberation. In figure 8, targets of varying reflectivity with respect to the background are used in simulations that are otherwise identical to the one used in figure 5(B). In figures 8(A)–(F), the target impedance mismatch was altered to yield reflectivity values ranging from 0.93 to 0.05, with all b-modes plotted on the same scale for this figure to allow for the visualization of reflectivity. Reflectivity is calculated by dividing the difference of the impedance values for the background and target by their sum. This is evident in figure 8(G), where reverberation curves preceding the target remain unchanged since the target reflectivity is the only variable.

In terms of overall image quality, a dim target, for example, will only be visible above the acoustic noise floor arising from reverberation, whereas a bright target will have higher contrast. This distinction is visible in figure 8(A) for a very bright target, in comparison to figure 8(F) for a target close to the noise floor. The impact of reverberation on image quality is thus *dependent* on target brightness. Conversely, the degradation from aberration is independent of target brightness because the phase distortion accumulated by the wave front on its path to the target is reflected by the point target. A brighter or dimmer target does not reflect more or less phase distortion. In other words, the impact of phase aberration on image quality is *independent* of target brightness. Indeed, the RMS value for phase aberration for a bright target (Figure 8(B)) was 133 ns and for a dim target (figure 8(E)) was 143 ns.

Trailing clutter appears due to the pulse lengthening of the wave that does not depend on the target brightness; however, its amplitude increases with increasing target reflectivity. Figure 8(G) shows how targets of high reflectivity produce high amplitude trailing clutter while low reflectivity targets produce lower amplitude trailing clutter.

#### 3.1.4. Anechoic lesion analysis

**Figure 9.**
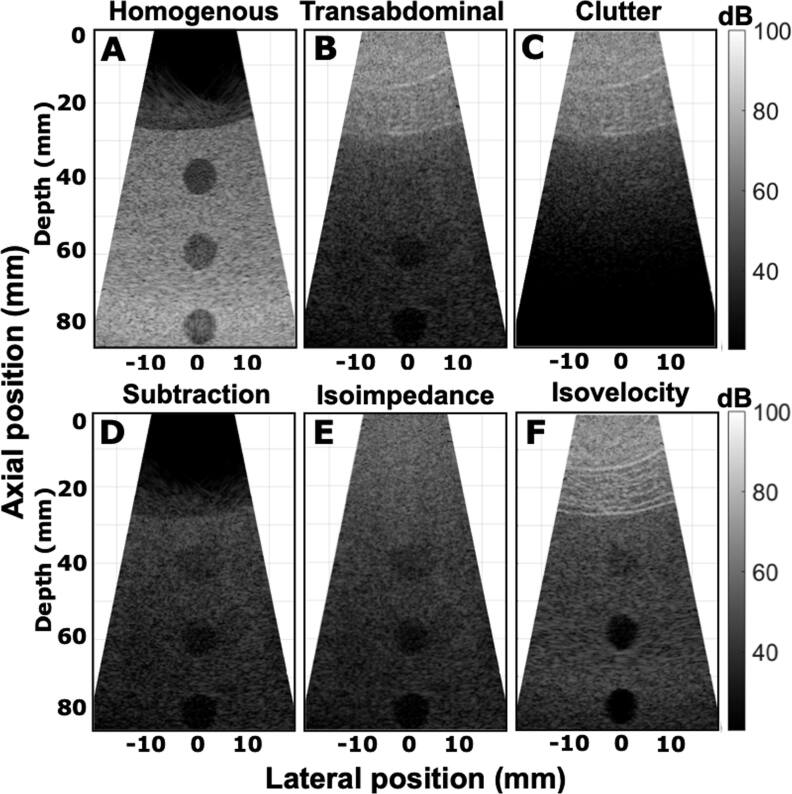
Anechoic lesions at 40, 60 and 80 mm, A for the homogeneous case, B for a transabdominal heterogeneous case, and C for the abdominal clutter in the absence of scatterers and anechoic lesions below. D, is the isoimpedance via subtraction of clutter B-mode. E, isoimpedance B-mode by setting the product of density and sound speed to be constant. F, isovelocity B-mode where the effects of aberration are removed and density is scaled to keep impedance constant. All lesions are reconstructed from 64 independent transmit-receive events focusing at the depth of the deeper lesion (80 mm) and following the curvature of the C5-2v array. All images are normalized with respect to the mean speckle value of the heterogeneous case. Units are in dB.

Even though PSFs accurately capture imaging capabilities, it can be hard to understand the clinical relevance based on PSF analysis alone. Anechoic lesions yield a more clinically relevant characterization of the imaging characteristics and degradation effects. For this purpose, the anechoic lesions described in section 2.6 were simulated and resulted in the B-mode images shown in figures 9 and 11.

**Figure 10.**
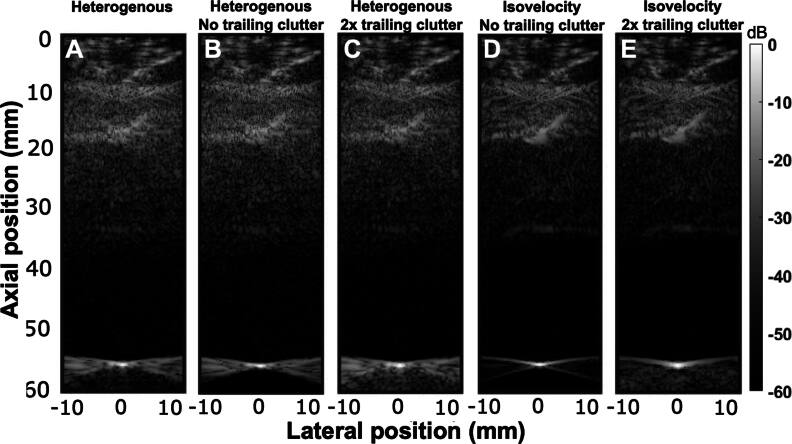
A, Original heterogeneous B-mode image, B, heterogeneous B-mode where the trailing clutter is removed and C, heterogeneous B-mode where the trailing clutter is re-introduced and increased by a factor of 2. D, isovelocity B-mode where the trailing clutter is removed and E, isovelocity B-mode where the trailing clutter is increased by a factor of 2.

First, a reference lesion image was established in the acoustic maps by inserting anechoic scatterer-free lesions in the randomly distributed sub-resolution scatterer medium (figure 9(A)). This is equivalent to an anechoic lesion phantom with a water path in the first ≈20mm. Then, the abdominal wall was inserted to simulate abdominal imaging, referred to as a heterogeneous case. This is the equivalent of embedding a body wall in the phantom. The contrast-to-noise ratio (CNR) for each of the three lesions was calculated Patterson and Foster (1983). The results are summarized in table 2, where ND stands for not detectable lesion. As illustrated in figure 9(B), the abdomen reduces both contrast and resolution in the image when compared to the scatterer-only case in figure 9(A). The top lesion, at 40mm, has the greatest reduction in image quality and does not appear to be detectable. This can be understood using figure 9(C), where there is a large amount of reverberation ring-down that appears beneath the abdominal wall. It is this reverberation that most significantly reduces the image quality immediately under the abdomen, reducing the CNR from 1.45 to not detectable (ND) (table 2). However, figure 9(C) also shows that the reverberation is significantly reduced as a function of depth. At a depth of 60 mm, the CNR for the scatterer-only case is 1.34 and for the abdominal case is 0.68. At a depth of 80 mm, the difference is even smaller with values of 1.30 and 1.00.

**Table 2.** CNR for the three lesions for different cases.

Type of emission	40 mm	60 mm	80 mm
Homogeneous	1.45	1.34	1.30
Heterogeneous	ND	0.68	1.00
Isovelocity	0.49	1.30	1.39
Isoimpedance	0.46	0.84	1.00
Isoimpedance by subtraction	0.39	0.78	1.00

In figure 9(C), only the body wall and no sub-resolution scatterers were included. Figure 9(D) did not require additional simulations and it is an image created by the subtraction of figure 9(C) from figure 9(B). In figure 9(E), an isoimpedance B-mode was generated using acoustic maps, where the product of sound speed and density was kept at a constant value of 1.67 MRayl, which is equal to the homogeneous value. This was achieved by scaling the density maps according to equation (3). Lastly, the isovelocity B-mode, shown in figure 9(F), was obtained by setting the speed of sound to be constant while scaling the density maps to preserve the original impedance value for the heterogeneous case (equation (5)). The isovelocity case restores almost all the image quality for the 60 mm and 80 mm lesions and the CNR for the scatterer-only and isovelocity images are almost the same. This indicates that the deep lesions are most affected by aberration. However the 40 mm lesion has a CNR of 0.49, which is significantly lower than the scatterer-only 1.45 CNR value. So, even though removing aberration improves the detectability of the shallow lesion, alone it is not sufficient to restore the image quality.

Figures 9(D) and (E) remove reverberation in different ways. Recall that the isoimpedance case (figure 9(E)) removes both multipath reverberation and trailing clutter. The reverberation subtraction case (figure 9(D)) removes multipath reverberation but retains trailing clutter. The CNR values for both cases are almost identical for all depths. This indicates that in this case, the effect of trailing clutter on image quality degradation is minimal. Removing reverberation partially restores the CNR value for the 40 mm lesion at a level that is comparable to phase aberration removal (0.46 vs 0.49, respectively). This indicates that for the shallow lesion, phase aberration and reverberation have a similar impact. However, for the 60 mm and 80 mm lesions, removing the effects of reverberation restores the image quality to a lesser degree than aberration. This is consistent with the observation that reverberation is depth-dependent but aberration is not.

### 3.2. Modulable reconstruction of image degradation

Having shown that image degradation components can be successfully isolated and characterized, it is demonstrated that selected degradation effects can be introduced in B-mode images in an independent manner. Degradation effects can be introduced in three ways, by modifying (a) the maps of the acoustic properties, (b) the received data, or (c) the transmitted emission. Here, we focus on the first two cases, since modifications of the transmit pulse, e.g., aberration or pulse lengthening, have been considered in prior work.

#### 3.2.1. Isolation and reconstruction of trailing clutter

In the PSF, isolation of the trailing clutter, which appears in the post-isochronous volume, can be achieved temporally in the RF data. Isolation of the trailing clutter was performed by first identifying the peak of the emission pulse and the target echo in the RF simulation data. The two signals were shifted so that their peaks temporally coincide, meaning that the tail of the echo signal, after the emission signal is equal to zero, corresponds to the lengthened trailing pulse. This is shown in figure 10, where the trailing clutter was removed from the heterogeneous B-mode in figure 10(A) by isolating the area following the point target, resulting in figure 10(B). Following its isolation, it can also be scaled and re-introduced back into the RF data of the image as shown in figure 10(C), where it has been scaled or amplified by a factor of 2.

**Figure 11.**
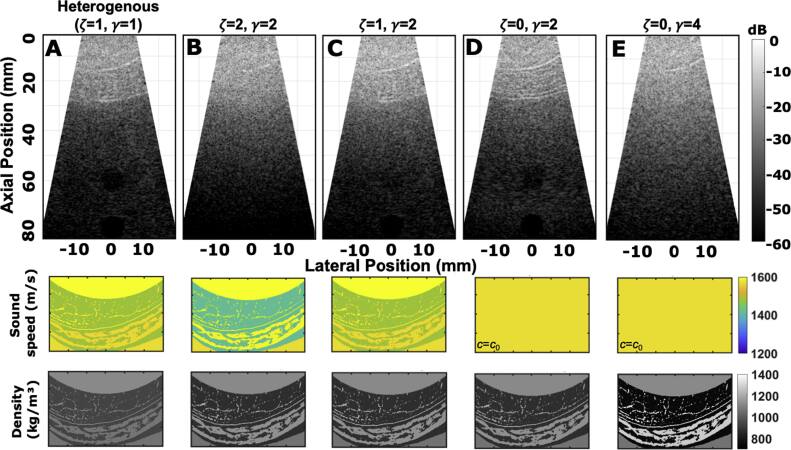
Anechoic lesion B-mode images for different configurations A-E (top row) and the respective sound speed (middle row) and density (bottom row) maps used to generate each of them, where sound speed and density are scaled. *ζ* scales the sound speed maps and *γ* scales the density maps based on equations (7)–(9). Constant speed maps are used for isovelocity B-modes where *c*_0_ is equal to a homogeneous 1570 m/s. A, the homogeneous case from the pre-scaled acoustic maps. B, the case where both sound speed and density are scaled by 2. C, the case where only density is scaled by a factor of 2. D, an isovelocity case where the sound speed is scaled by 0, or equivalent to *c*_0_ everywhere, and density is scaled by 2. E, an isovelocity case where density is scaled by 4.

Figures 10(A)–(C) all include the effect of aberration. However, it can be desirable to only include the effects of trailing clutter with no aberration. In this case, an isovelocity simulation can be performed and the trailing clutter can be removed, as shown in figure 10(D). Then, trailing clutter can be added in, as shown in figure 10(E), where it has again been scaled by a factor of 2. Figure 10(E) thus represents the case with no aberration and known and modulable amounts of trailing clutter. However, this process is only applicable to PSF imaging.

#### 3.2.2. Modulation of aberration and reverberation

Apart from the isolation and removal of reverberation and aberration from the image, it was also shown that aberration and reverberation can be artificially modulated by modifying the abdominal acoustic maps. A heterogeneous anechoic lesion B-mode is shown in figure 11(A). Then, aberration and reverberation were scaled in different combinations. Scaling was performed according to equations (7)–(9), using four different combinations of the parameters *ζ* and *γ*. Average acoustic values were used as equilibrium constants, i.e., *c*_0_ = 1570 m/s, *ρ*_0_ = 1064 kg/m^3^, and *Z*_0_ = 1.67 MRayl. In the first case (figure 11(B)), *ζ* = 2 and *γ* = 2, which increases both the aberration and reverberation. This modification completely removes the ability to image the anechoic lesion. The two bottom rows of figure 11 show a zoomed in version of the different levels of scaling of sound speed (second row) and density (third row) maps used to the B-mode image of each column A-E. Scaling the density and keeping the speed of sound map unchanged, *ζ* = 1 and *γ* = 2, has the effect of increasing the reverberation without modifying the aberration (figure 11(C)). In this case, the CNR of the 60 mm lesion is significantly reduced to 0.24, from 0.68 when compared to reference heterogeneous case (c.f. tables 2 and 3). For the deeper 80 mm lesion, there is a smaller reduction in CNR, to 0.77 from 1.00, which is consistent with the observation that post-abdominal reverberation clutter is depth-dependent. Scaling the density and homogenizing the sound map to its equilibrium constant, where *ζ* = 0 and *γ* = 2, has the effect of increasing the reverberation and removing the aberration (figure 11(D)). This improves the CNR for the lesions by approximately the same amount, to 0.48 for the 60 mm lesion, which is a difference of 0.24, and to 1.08 for the 80 mm lesion, which is a difference of 0.31 when compared to the *ζ* = 1 and *γ* = 2 case. It is also consistent with the observation that post-abdominally, aberration is not depth-dependent. Scaling the density even further while homogenizing the sound map to its equilibrium constant, so *ζ* = 0 and *γ* = 4, has the effect of further increasing the reverberation (figure 11(E)). At this level, the reverberation completely removes the ability to image the anechoic lesions in a way that is similar to the *ζ* = 2 and *γ* = 2 case.

**Table 3.** CNR for the three lesions for different cases of scaled maps.

Type of emission	40 mm	60 mm	80 mm
*ζ* = 2, *γ* = 2	ND	ND	ND
*ζ* = 1, *γ* = 2	ND	0.24	0.77
*ζ* = 0, *γ* = 2	ND	0.48	1.08
*ζ* = 0, *γ* = 4	ND	ND	ND

### 3.3. Augmentation of degradation effects

**Figure 12.**
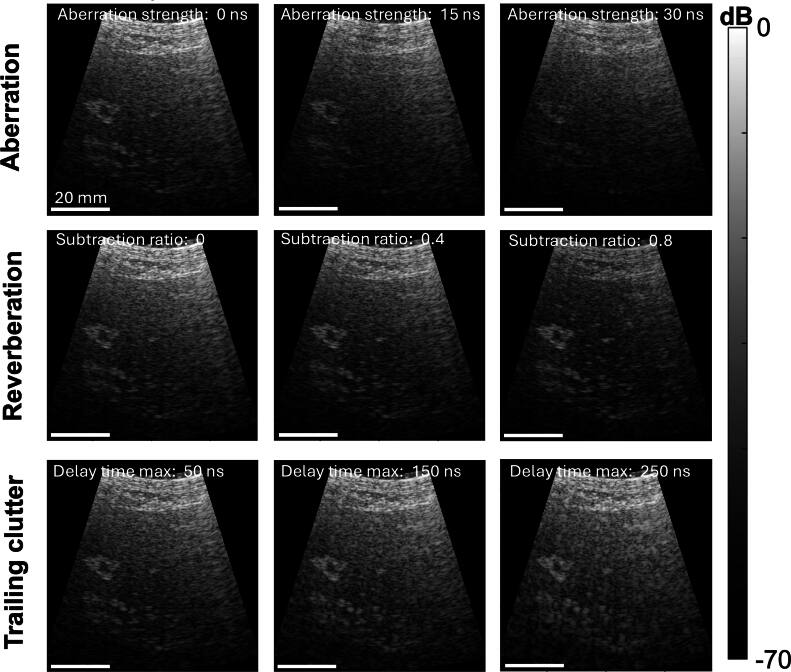
Reconstructed abdominal images created using the included code repository, where aberration (1st row) is augmented by phase screens with an RMS delay strengths between 0 ns RMS and 30 ns RMS, reverberation (2nd row) is augmented by subtracting the scaled abdominal wall signal from the heterogenous signal with a subtraction ratio between 0 and 0.8, and trailing clutter (3rd row) was applied using a depth-dependent damped sinusoid kernel with three maximum decay times ranging between 50 and 250 ns. All scalebars represent 20 mm.

While simulations can be performed to isolate and reconstruct these degradation effects in various combinations, these effects can also be modulated by known amounts in post-processing. This approach allows for faster generation of large datasets. An example of these post-processing augmentation techniques, applied to an abdominal dataset, are shown in figure 12, using the associated github repository described in the Appendix. These B-modes demonstrate augmentation of the three degradation effects with example aberration values ranging between 0 and 30 ns RMS, reverberation subtraction ratios between 0 and 1, and trailing clutter with maximum decay times between 50 and 250 ns. Based on this example dataset, which contains 128 transmit events, augmentation speeds were measured to be 9.5 × 10^−3^ s/sample for aberration, meaning that increasing 1000 data points by 100 times would take 0.26 hours. The augmentation speed was also measured to be 5.3 × 10^−3^ s/sample for reverberation and 6.6 s/sample for trailing clutter. With full synthetic aperture beamforming, as used in the example dataset, these speeds changed to 2.8 s/sample for aberration, 2.8 s/sample for reverberation, and 9.5 s/sample for trailing clutter.

## 4. Discussion and conclusion

Simulation tools offer the valuable ability to measure and study sound inside the body across different imaging scenarios. They can also be used to tractably modify acoustic properties and the resulting wavefield characteristics. To isolate image degrading effects into their individual components maps, acoustic maps were designed to remove aberration while preserving reverberation or to remove reverberation while preserving aberration. The components of medium-dependent image degradation were separated into three categories that affect an imaging pulse: (a) aberration of the phase and amplitude, (b) multiple scattering that is contemporaneous but of distal spatial origin (reverberation clutter), and (c) multiple scattering that is co-directional or spatially proximal to the imaging pulse (trailing clutter).

It was shown that the Fullwave2 simulation tool, which uses a propagation physics based approach, can closely model the acoustics in the abdominal wall. Comparisons of porcine body wall and liver experiments to simulations in visible human maps of the body wall and liver showed that a close match can be achieved. The aberration for the experiment was calculated at 143.7 ns, whereas for the simulations, the RMS value was calculated at 146.0 ns. Reverberation ringdown curves were either super-imposed or within 1-2 dB for all considered depths from 0 to 55 mm, over a range of 0 to -55 dB. Lag-one coherence, which is a spatial coherence based metric of image quality, was measured to be 0.95 for the experiment and 0.96 for the simulation. The liver does also generate its own clutter, but it is weak compared to the abdominal wall. The speckle brightness values from the liver was measured to be −43.0 dB experimentally and −43.2 dB *in silico*. This established confidence that the parameter ranges for the acoustic maps created from the human tissue maps were relevant to the image quality metrics and analysis that was subsequently performed. This study was performed in 2D, but future work using 3D Fullwave2 (https://github.com/gfpinton/fullwave2_3d_public.git) can provide further insight into these acoustic phenomena.

The isolation into the three separate categories was used to evaluate the effect of target brightness and depth. According to the physical model, one would predict that image quality is unaffected by target brightness for aberration and trailing clutter but it is affected for multiple reverberation. It was shown that changing the target brightness will increase the target signal magnitude compared to the reverberation signal magnitude. It was also shown that changing the target brightness proportionally increases the magnitude of the reflected imaging pulse, which includes the trailing clutter component. In other words, trailing clutter magnitude scales with target brightness and reverberation clutter does not. Consequently, the effects of reverberation clutter on image quality depend on target brightness and the effects of trailing clutter on image quality do not depend on target brightness. It was also shown that post-abdominally, the reverberation magnitude decays, whereas trailing clutter remains constant as a function of depth.

The aberration magnitude was measured to be 143, 130, and 139 ns at depths of 45, 55, and 65 mm, respectively. Reverberation acts as a confounding factor in the measurement of phase aberration since it adds acoustic ‘noise.’ These measurements were thus performed in simulations that had the reverberation removed. These RMS values align with previous studies showing that aberration is depth independent Soulioti *et al* (2025), Pinton *et al* (2011). However, there are small differences in the RMS values at the three different depths, which could require more investigation in future studies. Using these simulations, it was also shown that, as expected, target brightness did not affect the reverberation estimates.

To measure isoimpedance, clutter subtraction simulations were used, which do maintain trailing clutter due to the absence of a point target in the clutter simulation used for subtraction. This clutter subtraction method was used due to the presence of residual reverberation clutter in isoimpedance simulations. This is likely due to small numerical discretization errors at material interfaces on the staggered grid, which give rise to spurious reflections. These reflections are a limitation in many numerical methods, so the clutter subtraction approach was developed to address this. Future work to better characterize this problem is required, including simulations using Fullwave 2.5 Sode and Pinton (2025), which is capable of modeling a broad range of power-law attenuation models, improving attenuation estimates.

Even though the PSF characterization is useful to determine relative changes in energy distributions and their space-time locations, it implies a linear shift invariant system, which is not without its limitations in terms of interpreting the sources of image degradation from the distribution of energy within the PSF. For example, the energy within the isochronous volume is large but confined to a relatively small volume. Reverberation clutter, on the other hand, has low energy, but is distributed over a much larger volume. Furthermore, the extent of this volume depends on the reverberation time constants, which are material-dependent. Long reverberation time constants yield a spatially larger reverberation PSF. In a convolutional framework, the energy over the entire extent of the PSF must be determined so that it can be integrated into the representation of a point in an image. In other words, the low energy but wide spatial extent of reverberation places additional limitations on the PSF paradigm. Therefore, since they do not rely on linear shift invariance assumptions, B-mode images provide a valuable additional tool to characterize these degradation mechanisms.

An anechoic lesion model of B-mode imaging was thus established to determine the impact of these aberration and reverberation characteristics on image quality. The separate contributions of aberration, multiple reverberation clutter, and trailing clutter to image quality was determined using combinations of scatterer maps, abdominal maps, isovelocity maps, and isoimpedance maps. This analysis was performed as a function of depth. The CNR analysis for this particular abdominal model showed that shallow lesions are more affected by reverberation and deep lesions are more affected by aberration. For the anechoic lesion cases, trailing clutter has a smaller impact on image quality; however, highly reflective targets, like those shown in figure 8, can produce high amplitude trailing clutter. However, different tissue maps, transducer configurations, transmit frequencies, imaging, and beamforming methods may yield differences in these dependencies. The proposed simulation methods framework a way of determining and quantifying which source of degradation is relevant.

Furthermore, apart from the isolation and removal of reverberation and aberration from the image, it was also shown that this simulation framework offers the opportunity to controllably modulate the amounts of reverberation and aberration by modifying the acoustic maps. Even non-realistic scenarios of, for example, four times denser abdominal layers, can be explored and the effects of image degradation can still be isolated and quantified. This flexibility along with the ability to simulate a variety of anatomical structures may offer significant benefits for the design and testing of beamforming approaches.

In addition to the simulation approaches to isolate and modulate these effects *in silico*, these effects can also be artificially modulated in post processing on any dataset, as shown in the associated github repository described in the Appendix. This code repository can be used to generate large volumes of data without the need to re-run multiple simulations. This can be used for purposes of algorithm training in machine learning applications with known amounts of image degradation and known tissue maps, something that cannot be achieved clinically where the ground truth is unknown. This can also be used for algorithm assessment under known magnitudes of different degradation effects. Since these are post processing techniques, they make some assumptions. The trailing clutter case implements depth-dependent trailing clutter but, due to fast computational speed requirements for augmentation, it does not incorporate spatial variability. The reverberation technique modulates clutter values based on the heterogeneous signal, but this may also scale some aberration effects present in the underlying dataset. Both of the reverberation techniques scale clutter, so do not create new realizations of the clutter. Aberration is applied using a receive-side phase screen instead of two-way propagation. However, these techniques are intended to be close estimates for post-processing augmentation. If full separation and multiple realizations of degradation effects are necessary, the full simulation framework described in the rest of the paper is required, with a different simulation for each case.

The example augmentation included here is based on Fullwave2 simulations. However, any ultrasound simulation can be used. These augmentation approaches can also be used on experimental datasets, but the reverberation case requires an estimation of clutter. This augmentation approach allows us to increase 1000 datapoints by 100 times in 0.26 hours for the aberration, 0.14 hours for reverberation, and 183 hours for depth-dependent trailing clutter. The original simulation consists of 128 transmit events, each of which take a few minutes to run on a GPU, so this augmentation approach is much faster than running simulations for different combinations of degradation parameters. This augmentation was performed in python while the reconstruction and deconstruction of effects were performed in MATLAB. Fullwave2 simulations can also be performed in python using the following simulation repository: https://github.com/pinton-lab/fullwave_python.git.

In conclusion, it is shown that reverberation is depth and brightness dependent, while aberration and trailing clutter are not. In addition, we have demonstrated how individual components of image degradation, including aberration, reverberation, and trailing clutter, can be reversibly and linearly isolated, quantified, and modulated in B-mode imaging through the human abdomen using full-wave propagation simulations, such as Fullwave2. Furthermore, an ultrasound framework to generate large augmented datasets incorporating wide physics-based ranges of image degradation is presented that can be used with machine learning models.

## Data Availability

The data that support the findings of this study are openly available at the following URL/DOI: https://doi.org/10.17615/zmxs-3741.

## Appendix Code repository for augmented image degradation dataset creation

In addition to the ability to be isolated and reconstructed *in silico*, aberration, reverberation, and trailing clutter can be artificially augmented in post processing for any simulation or experimental dataset. This allows for the creation of larger datasets with known and varying degradation values for machine learning applications. The code to generate these augmented images in post-processing to any dataset, written in python, is publically available at the following repository: https://github.com/pinton-lab/us_degrade_reconstruction.git. Example cases with differing levels of aberration, reverberation, and depth-dependent trailing clutter were applied to previously published Fullwave2 simulations using a publically available abdominal dataset on an Intel(R) Core(TM) i9-13900K CPU Zhuang *et al.* (2025). Reverberation was augmented by scaling the abdominal wall signal and subtracting it from the heterogeneous simulation. Aberration was augmented by applying a spatially varying phase screen with different RMS delay strengths. Trailing clutter was applied using a depth-dependent damped sinusoid kernel with different decay times via convolution.
